# Role of diet, physical activity and new drugs in the primary management of cancer cachexia in gastrointestinal tumors – a comprehensive review

**DOI:** 10.3389/fonc.2025.1600425

**Published:** 2025-05-30

**Authors:** Ester Oneda, Alessandra Manno, Silvia Noventa, Michela Libertini, Sara Cherri, Alberto Zaniboni

**Affiliations:** Department of Clinical Oncology, Fondazione Poliambulanza, Brescia, Italy

**Keywords:** cancer cachexia, gastrointestinal tumors, nutritional support, physical activity, new drugs, multimodal approach

## Abstract

Cancer cachexia is a multifactorial syndrome characterized by involuntary weight loss, muscle mass reduction, and systemic inflammatory response, negatively impacting the quality of life and survival of cancer patients. In gastrointestinal tumors, cachexia prevalence can reach up to 80%, presenting a complex clinical challenge. This review aims to explore the efficacy of a multimodal approach integrating nutritional support and physical activity in cancer cachexia management. Targeted dietary strategies, such as a high-protein diet enriched with omega-3 and branched-chain amino acids (BCAAs), have proven effective in counteracting muscle loss and modulating inflammatory metabolism. Physical activity, particularly resistance training, contributes to preserving lean mass and improving physical function. However, current data suggest that no single strategy is sufficient to reverse cachexia, necessitating combined treatment with supportive pharmacological therapies, including progestogens, muscle metabolism modulators, and cytokine antagonists. Despite the evidence supporting these interventions, significant gaps remain, highlighting the need for randomized clinical trials to define the optimal therapeutic protocol for gastrointestinal cancer patients with cachexia.

## 1 Introduction

Malnutrition is a pathological condition characterized by insufficient nutrient intake, metabolic imbalances, or inadequate utilization of nutrients by the body. This leads to a reduction in body mass, muscle function, and immune response. When, in addition to reduced nutrient intake, there is metabolic dysregulation and chronic systemic inflammation, muscle mass loss becomes irreversible with nutritional support alone. At this stage, malnutrition progresses into cachexia. This process is mediated by pro-inflammatory cytokines such as IL-6, TNF-α, and IL-1β, which stimulate muscle degradation and the consumption of lipid stores, thereby altering the body’s energy balance (1).

Cancer cachexia is a multifactorial syndrome characterized by involuntary weight loss, mainly due to a reduction in skeletal muscle mass, often accompanied by a decrease in fat mass (2). This condition is highly prevalent among cancer patients, especially those with gastrointestinal tumors, where prevalence can reach up to 80% during the course of the disease. Cachexia results from a combination of factors, including the direct metabolic effects of the tumor, appetite loss, and reduced nutrient intake. This leads to significant muscle mass loss (sarcopenia) and reduced tolerance to oncological treatments. The progression of cachexia occurs in three stages: pre-cachexia (weight loss <5% over the past 6 months), cachexia (weight loss >5% over 6 months, or >2% with sarcopenia), and refractory cachexia (reduced individual autonomy [PSECOG] and advanced clinical deterioration) (2).

Several studies have shown that malnutrition is associated with increased toxicity of oncological treatments, higher risk of hospitalization, and reduced therapeutic response (3). Moreover, malnourished patients have lower overall survival, with a six-month survival rate of 47% compared to 84% in non-malnourished patients (p = 0.0003) (3). Malnutrition and physical inactivity are highly prevalent in cancer patients undergoing experimental treatments, with prevalence rates of 40% and 50%, respectively (3). Malnourished patients exhibit shorter durations in clinical trials and a higher incidence of grade ≥3 adverse events (3). Furthermore, a sedentary lifestyle has been associated with reduced treatment adherence (3, 4).

Therefore, thorough nutritional and physical assessments are essential before initiating oncological therapies to improve treatment tolerance and optimize overall clinical outcomes. Given that the mechanisms of malnutrition involve chronic inflammation and negative energy balance, adopting a multimodal approach that combines pharmacological, nutritional, and physical treatments is crucial. In recognition of the need for proper management of this complication, guidelines have been developed by international and national institutions such as NCCN and ESMO (5, 6).

The objective of this review is to analyze the role of nutrition and physical activity in the primary management of cancer cachexia in patients with gastrointestinal tumors, with a dedicated section on supportive therapies and artificial nutrition.

## 2 Pathophysiology of cancer cachexia in gastrointestinal tumors

Cancer cachexia is characterized by an imbalance between anabolism and catabolism, exacerbated by metabolic alterations and systemic inflammation leading to muscle protein degradation. In cancer patients, the balance between pro-inflammatory and anti-inflammatory processes is disrupted, resulting in impaired immune activation and suppression. Cytokines not only influence body tissues but also act within the tumor microenvironment, contributing to systemic inflammatory responses.

### 2.1 Triggering factors

#### 2.1.1 Pro-inflammatory cytokine secretion

Cytokines play a crucial role in tumor processes, including initiation, progression, invasion, and metastasis. Previous studies have demonstrated a correlation between cytokine production levels and the prevalence of cachexia in certain tumor types. Key cytokines implicated in cancer cachexia include IL-6, TNF-α, TGF-β, INF-γ and MIC-1/GDF15. MIC-1/GDF15, a member of the TGF-β superfamily, is secreted in large quantities by both normal and tumor cells. It influences hunger centers in the hypothalamus and brainstem, leading to anorexia and subsequent cachexia (7). Beyond its dual role as a tumor suppressor and activator, TGF-β has emerged as a key metabolic regulator. Through the SMAD2/3 signaling pathway, it promotes muscle catabolism by activating mechanisms related to myostatin, which inhibits protein synthesis and enhances protein degradation. Myostatin (GDF8) is a well-known negative regulator of muscle mass. Additionally, TGF-β is involved in weight loss, muscle atrophy, and fibrosis, highlighting its significance in cancer-related metabolic dysregulation (7). Other cytokines, such as interleukin-6 (IL-6), TNF-α, INF-γ and IL-1β, contribute to muscle and adipose tissue loss by promoting proteolysis and lipolysis (8). Tumor necrosis factor-alpha (TNF-α) is a pro-inflammatory cytokine capable of inducing cachexia in murine models. It contributes to muscle atrophy by promoting muscle protein degradation through the activation of ubiquitin ligase genes, leading to the destruction of myofibrillar proteins and key transcription factors. However, clinical studies employing anti-TNF-α antibodies in cancer patients with cachexia have failed to reverse muscle atrophy, suggesting the need for additional anti-inflammatory strategies (8, 9). IFN-γ is capable of inducing weight and muscle loss and promoting inflammation, particularly in experimental models. However, its role may be less dominant compared to other cytokines like IL-6. Interleukin-6 (IL-6) collaborates with TNF-α or acts independently to mediate systemic inflammation in cancer cachexia. Elevated IL-6 levels are associated with weight loss and reduced survival in cancer patients (9). Although IL-6 can induce muscle atrophy, this effect is primarily observed at supraphysiological doses. Clinical trials with anti-IL-6 monoclonal antibodies have shown some symptom improvements, such as reduced anorexia and fatigue, but have not demonstrated significant impacts on lean mass loss. Growth Differentiation Factor 15 (GDF15) is strongly associated with cancer cachexia, as it promotes weight loss through anorexia in animal models. It regulates the anorexia-cachexia syndrome by interacting with the GFRAL receptor expressed in the central nervous system. Preclinical studies have shown that antibodies against GDF15 and GFRAL effectively counteract weight loss in tumor-bearing mice, suggesting their potential as therapeutic targets for cancer cachexia (10). Some chemotherapeutic agents can induce elevated GDF15 levels, leading to anorexia, nausea, and vomiting, effects that may be exacerbated in patients with reduced glomerular filtration (11).

#### 2.1.2 Increased lipolysis and basal metabolism

Cachectic patients exhibit a reduced response to anabolic stimuli, where the normal process of muscle protein synthesis is compromised due to factors such as surgical interventions, trauma, chronic debilitating diseases, aging, sedentary behavior, and corticosteroid use. Tumor cells produce Proteolysis-Inducing Factor (PIF), which inhibits muscle protein synthesis and accelerates protein degradation through caspase activation, promoting cellular apoptosis (12). Under pro-inflammatory stimulation, muscle proteins are degraded via the ubiquitin-proteasome system and autophagy, contributing to insulin resistance and increasing hepatic gluconeogenesis, thereby hindering glucose and amino acid absorption by muscles and exacerbating protein catabolism and cachexia progression. The activation of hormone-sensitive lipase (HSL) and reduced activity of lipoprotein lipase (LPL) lead to increased mobilization of lipid reserves, contributing to weight and fat mass loss. Additionally, the release of Lipid-Mobilizing Factor (LMF) by tumor cells stimulates lipolysis by converting triglycerides into fatty acids (13). Under the influence of cytokines such as IL-6 and parathyroid hormone-related protein (PTHrP), white adipose cells express UCP1 (uncoupling protein 1), which stimulates thermogenesis and increases energy dissipation as heat, worsening the energy deficit. Experiments in murine cachexia models have shown that suppressing inflammatory cytokines reduces UCP1 expression and prevents weight loss despite tumor progression (14). Furthermore, proliferin-1, a protein secreted by tumor cells, inhibits the formation of new adipocytes and stimulates lipolysis, accelerating adipose tissue depletion.

#### 2.1.3 Gastrointestinal dysfunction

Gastrointestinal tumors often present with symptoms such as diarrhea or steatorrhea, nausea, vomiting, dysmotility, and pain related to stenosis or perforations. Malabsorption leads to reduced availability of essential nutrients and weight loss, worsening cachexia (15). Oncological treatments themselves may contribute to persistent malabsorption, necessitating individualized treatment and nutritional support strategies (16).

#### 2.1.4 Loss of appetite

Chronic inflammation significantly impacts the hypothalamic-pituitary-adrenal (HPA) axis, altering energy metabolism regulation and inducing a negative energy balance. This inflammatory state increases resting energy expenditure (REE), resulting in disproportionate energy consumption relative to caloric intake, causing anorexia. The expression of pro-inflammatory cytokines in the hypothalamus inhibits NPY/AgRP neurons and activates POMC/CART neurons (2, 17). This imbalance suppresses appetite and contributes to progressive weight loss. Anorexia may also be aggravated by physical symptoms such as pain, fever, diarrhea, abdominal discomfort, and respiratory difficulties, as well as psychiatric disorders like depression and delirium. Recently, interest has grown in ghrelin, a hormone secreted by the stomach that stimulates appetite. Ghrelin not only promotes food intake but also exerts anti-inflammatory effects, inhibits muscle degradation via MuRF-1/MAFbx pathways, enhances muscle protein synthesis through IGF-1, reduces cellular apoptosis, increases lipid reserves, and lowers energy expenditure. Consequently, ghrelin is considered a potential therapeutic target for anorexia associated with cancer cachexia (18). Another hormone involved in appetite regulation is leptin, secreted by adipose tissue, which acts on hypothalamic receptors to suppress hunger. In cancer cachexia patients, leptin receptor levels are elevated, intensifying the hormone’s anorexigenic effect and further exacerbating weight loss (19).

## 3 Role of diet in cancer cachexia

Diet plays a crucial role in managing cancer cachexia. Although no nutritional strategy alone can reverse cachexia, dietary intervention represents an essential pillar of a multimodal approach aimed at improving clinical outcomes in patients.

### 3.1 Nutritional goals

The primary objective of nutritional support in cancer patients with cachexia is to counteract weight loss and preserve muscle mass, thereby enhancing quality of life and tolerance to oncological treatments. Targeted dietary supplementation can modulate metabolism, reduce inflammation, and support the patient’s energy requirements (13, 20).Maintaining nutritional status is critical to preserving muscle mass and reducing sarcopenia, thus limiting the negative impact of cachexia on oncological treatments (15, 21). A well-nourished patient exhibits better treatment responses, optimized chemotherapy tolerance, and reduced side effects such as fatigue and drug toxicity (20, 22). Additionally, nutritional status is vital for immune system efficiency, reducing infections and enhancing healing capacity (23). Finally, maintaining or improving nutritional status improves quality of life by alleviating symptoms like asthenia, appetite loss, and energy deficits (24).

### 3.2 Macronutrients and nutritional strategies

To achieve these objectives, nutritional support is based on a high-protein and high-calorie diet, combined with supplementation to counteract muscle degradation and modulate inflammatory metabolism (25, 26). Moreover, a multidisciplinary approach integrating diet, physical exercise, and pharmacological therapies is essential to improve the overall clinical outcomes of the patient (20, 24).

#### 3.2.1 Proteins

In cancer patients with cachexia, the recommended protein intake ranges between 1.2 and 2.0 g per kg of body weight per day. Certain nutrients, such as creatine and carnitine, may contribute to maintaining muscle mass, as suggested by some studies (26). Branched-chain amino acids (BCAAs)—leucine, isoleucine, and valine—play an interesting role. Studies conducted on animal models of cachexia have demonstrated their capacity to counteract proteolysis and prevent muscle atrophy (27). Moreover, phase II clinical studies have shown that oral administration of BCAAs, at doses ranging from 10 to 20 g per day, can improve anorexia in cancer patients. This effect is believed to be linked to their capacity to reduce tryptophan entry into the brain, thereby decreasing serotonin synthesis, which is associated with reduced appetite (28). However, the efficacy of BCAAs in treating cancer cachexia remains unclear. Some research suggests that their supplementation may not be sufficient to counteract the effects of chemotherapy, which tends to interfere with BCAA metabolism, reducing their potential benefit in preserving muscle mass (29–31). Furthermore, there are indications of a possible gender difference in response to cachexia and BCAA metabolism: for instance, women may be more vulnerable to chemotherapy-induced muscle mass loss compared to men, suggesting the need for personalized approaches (30). Overall, while some studies have reported promising results, others have not demonstrated a significant impact of BCAAs on the progression of oncological cachexia, highlighting the need for further research to fully understand their role and optimal use (32, 33). Another nutritional approach investigated for cancer cachexia involves the combination of beta-hydroxy-beta-methylbutyrate (HMB), glutamine, and arginine. However, in a randomized, placebo-controlled phase III clinical trial (RTOG 0122 study), this strategy did not prove effective in increasing lean mass or improving patients’ quality of life. Moreover, the study reported a high dropout rate: only 37% of patients completed the protocol, and 45% were lost to follow-up (34). Consequently, there is currently insufficient and inconsistent data to recommend the use of these agents in cancer cachexia treatment.

#### 3.2.2 Fatty acids and omega-3

Medium-chain triglycerides (MCTs) and omega-3 fatty acids play a significant role in managing cancer cachexia due to their ability to modulate metabolic processes to the host’s advantage.

##### 3.2.2.1 Medium-chain triglycerides

MCT-rich diets can reduce weight loss in cancer cachexia models. In a murine model with colon adenocarcinoma, a diet in which 80% of energy came from MCTs reduced body weight loss, promoting an increase in both fat and lean mass (35). MCTs elevate ketone body levels, such as acetoacetate and 3-hydroxybutyrate, providing a crucial energy source during starvation, which tumor cells find difficult to utilize. Additionally, MCTs have been shown to reduce circulating free fatty acid (FFA) levels without significantly altering blood glucose or insulin levels, suggesting a favorable metabolic effect for the host (35).

##### 3.2.2.2 Omega-3 fatty acids

Omega-3 fatty acids, particularly EPA (eicosapentaenoic acid) and DHA (docosahexaenoic acid), are known for their anti-inflammatory properties and potential to improve nutritional status in cancer cachexia patients. Omega-3s reduce inflammation by inhibiting the production of inflammatory cytokines and catabolic factors that contribute to muscle protein degradation (36). They promote muscle protein anabolism and inhibit proteolysis, helping to counteract muscle loss (37). Studies have shown that omega-3 supplementation can improve body weight, quality of life, and reduce cachexia indices, particularly in patients with advanced non-small cell lung cancer (38).

##### 3.2.2.3 Fish oil

Clinical studies on fish oil have shown variable benefits. Supplementation with EPA and DHA in a 2:1 ratio, at minimum doses of 2 g/day for at least six weeks, has been associated with improvements in lean muscle mass, chemotherapy tolerance, and quality of life, although significant benefits on overall survival have not always been observed (39). Benefits appear to depend on tumor type, composition, and duration of supplementation.MCTs and omega-3s represent promising interventions in managing cancer cachexia, but their efficacy varies depending on tumor type and dosage. Further studies are necessary to optimize dosing and evaluate their impact on quality of life and clinical outcomes. A personalized therapeutic approach may be essential to maximize the benefits of these supplements.

#### 3.2.3 Carbohydrates

Although sugar itself is not directly implicated in cancer or cachexia onset, altered glucose metabolism and insulin resistance associated with cachexia suggest that managing dietary components, including sugar, could be part of a broader strategy to address metabolic dysfunctions in these patients. Nutritional interventions focus on high-energy diets and managing insulin resistance; particular attention is given to tumors like pancreatobiliary adenocarcinoma, which significantly impair insulin regulation. This context introduces the Warburg effect, which refers to the preference of cancer cells to metabolize glucose anaerobically, converting it into lactate even in the presence of oxygen. This metabolic reprogramming is a distinctive feature of cancer cells and is linked to increased glucose uptake and glycolysis, processes that support rapid proliferation and survival (40–43). The Warburg effect leads to an increase in glycolytic flux and lactate production, creating a tumor microenvironment that promotes tumor growth and metastasis while contributing to systemic effects like cachexia (43). Targeting the Warburg effect through glycolytic inhibitors has shown potential in reducing tumor-induced cachexia and improving energy balance in experimental models (44, 45). However, no efficacy has been demonstrated for low-carbohydrate nutritional strategies. Consequently, a balanced dietary regimen that avoids excess refined sugars is recommended to mitigate insulin resistance.

### 3.3 Specific dietary strategies

#### 3.3.1 Mediterranean diet

The Mediterranean diet appears to have a positive impact on survival and quality of life in cachectic neoplastic patients, particularly those with colorectal cancer. This diet, rich in anti-inflammatory and antioxidant nutrients, can improve body composition, reduce inflammation, and enhance overall health outcomes in cancer patients. A study found that patients with colorectal cancer-induced cachexia who adhered to a Mediterranean diet experienced significant improvements in weight, lean body mass, fat mass, and muscle strength compared to those receiving standard nutritional counseling (46).The Mediterranean diet was associated with decreased levels of inflammatory markers such as TNF-alpha, hs-CRP, and IL-6, which are crucial in managing cachexia and improving patient outcomes (46). Patients following the Mediterranean diet reported better global health status and physical performance, indicating an overall improvement in quality of life (46). Adherence to the Mediterranean diet has been linked to lower all-cause mortality in cancer survivors, including those with colorectal cancer, suggesting a potential benefit in extending survival (47–49). For patients undergoing chemotherapy, the Mediterranean diet has shown to reduce cancer-related fatigue, particularly in those with initially low adherence to the diet (50).The Mediterranean diet offers several benefits for cachectic neoplastic patients, including improved body composition, reduced inflammation, and enhanced quality of life. These benefits may contribute to better survival outcomes, making the Mediterranean diet a valuable dietary strategy in managing cancer cachexia.

#### 3.3.2 Ketogenic diet

Ketogenic diets (KDs) have been studied in managing cachexia in cancer patients, particularly for preserving muscle mass and functionality, although results are mixed and further research is needed. KDs may help preserve muscle mass and improve muscle function in cancer models, such as pancreatic tumors, by maintaining greater muscle mass and grip strength compared to standard diets (51, 52). Moreover, KDs have been shown to reduce systemic inflammation, potentially preventing muscle loss and weight reduction (53). The goal of KDs is to alter tumor cell metabolism by reducing glycolytic flux and glutamine uptake, leading to decreased tumor growth and cachexia. However, while KDs may delay tumor growth, they can accelerate cachexia onset in some tumor models due to metabolic imbalances, such as corticosterone deficiency (54).One study suggests a promising role for KDs combined with gemcitabine, attenuating cachexia and preserving muscle mass (52). However, KD efficacy may vary based on tumor type and individual metabolic responses, emphasizing the need for personalized dietary interventions (55, 56). Furthermore, adherence to ketogenic diets can be challenging, with some patients experiencing significant weight loss and mild to moderate side effects (57, 58). Current studies often present methodological limitations and heterogeneous results, highlighting the need for further controlled clinical trials to better understand KD mechanisms and efficacy in cancer treatment (57, 58).

#### 3.3.3 Nutraceuticals and supplements

##### 3.3.3.1 β-hydroxy-β-methylbutyrate

HMB has been studied as a dietary supplement for cancer patients, particularly in the context of tumor cachexia. HMB has demonstrated its ability to attenuate muscle mass and body weight loss in experimental models of cancer cachexia. Studies in rats have shown that HMB supplementation increases muscle and cardiac weight and prevents body weight loss, suggesting an improvement in protein anabolism in muscle tissues (59).

HMB is believed to support muscle mass preservation by activating signaling pathways that enhance protein synthesis and suppress protein degradation, making it a potential component of multimodal therapies against tumor cachexia (60). A systematic review found evidence suggesting beneficial effects of HMB on muscle mass and function in cancer patients, although the effects on quality of life and body weight were less clear. The review highlighted the need for well-designed studies to further explore these benefits (61). Clinical evidence remains conflicting, and further research is necessary to confirm the efficacy and safety of HMB in cancer patients.

##### 3.3.3.2 Probiotics

Probiotics may offer significant benefits in cancer patients with cachexia by improving gut health and reducing systemic and intestinal inflammation. Their beneficial effects include restoring intestinal barrier functionality, often compromised during chemotherapy due to systemic and localized inflammation. In patients with gastrointestinal tumors, the use of multi-strain probiotics combined with prebiotics like inulin is recommended to reduce the risk of infections and intestinal inflammation (ESPN) (62). In cachectic patients, the gut microbiome undergoes significant changes, with a reduction in beneficial bacteria such as Clostridiales and Lactobacillus and an increase in Bacteroidetes and Enterobacteriales (63). Supplementation with Lactobacillus strains in animal models has shown benefits in reducing muscle atrophy and systemic inflammation markers (64). The probiotic Lactobacillus rhamnosus has demonstrated, in animal models, the ability to enhance the efficacy of capecitabine, reducing tumor size, protecting white blood cells, and decreasing systemic inflammation (IL-6). Clinical studies in patients with colorectal cancer treated with 5-fluorouracil have reported fewer episodes of diarrhea, abdominal pain, and hospitalizations in the probiotic group (65). Probiotic use may also be applied preventively to reduce the onset of gastrointestinal side effects from chemotherapy, such as diarrhea and malnutrition, by stabilizing intestinal function and improving nutrient absorption. For instance, the probiotic mixture VSL#3 has been shown to reduce irinotecan-induced intestinal toxicity, prevent weight loss, and promote intestinal crypt proliferation (66). Moreover, patients treated with probiotics for six months post-surgery showed significant reductions in inflammatory biomarkers (TNF-α, IL-6, IL-10, etc.) and improved quality of life. These benefits were particularly evident in patients with good functional status, indicating that probiotics may prevent cachexia progression by improving appetite, body weight, and nutritional status (67).

#### 3.3.4 Oral nutritional supplements

Oral nutritional supplementation (ONS) is widely used to support patients with cancer cachexia, aiming to improve nutritional status, body composition, and quality of life. ONS are supplements enriched with specific nutrients (e.g., eicosapentaenoic acid, HMB/arginine/glutamine, omega-3 fatty acids, micronutrients, and probiotics) that have shown particular benefit in stabilizing or increasing body weight and fat-free mass, even in advanced cancer (68–70). When combined with dietary counseling, significantly increases protein and energy intake, improves nutritional status, and can lead to clinically meaningful gains in weight and lean body mass in patients with cancer cachexia (68–72). ONS interventions are associated with improvements in quality of life, functional capacity, and performance status, as well as better tolerance to chemotherapy and radiotherapy (69–71, 73, 74) with fewer interruptions or delay of planned treatments due to toxicity or malnutrition (70, 73). Although ONS alone are often insufficient to reverse cachexia, they remain a fundamental component of multimodal treatment.

## 4 Physical activity and muscle mass preservation

Physical exercise represents an interesting strategy for managing cancer cachexia. Given the muscle-metabolic etiology, physical activity can be proposed synergistically with nutritional and pharmacological treatments to counteract the detrimental effects of cachexia. The benefits of physical exercise aim to improve muscle metabolism and reduce inflammation. Specifically, strength training and combined resistance-aerobic exercises help counteract muscle loss by promoting protein synthesis and reducing muscle protein degradation (24, 75). Furthermore, physical activity helps reduce inflammation, potentially limiting muscle catabolism (75, 76). Regular physical activity can improve physical functionality, muscle strength, and quality of life in cancer patients, often compromised due to cachexia (24, 75, 77). Moreover, exercise could help mitigate cardiac dysfunction and respiratory muscle atrophy associated with cachexia, promoting better general health and greater tolerance to oncological treatments (77).

### 4.1 Effects of physical exercise

#### 4.1.1 Muscle mass and strength

A primary objective of exercise in cachexia is to preserve or increase muscle mass (lean body mass) and improve strength. A systematic review in 2023 (12 controlled studies, including 9 RCTs) reported that approximately 75% of examined outcomes showed a positive trend in body weight or composition (lean/fat mass) in patients who engaged in exercise (78). In most studies, exercise helped stabilize weight or limit weight loss compared to standard care. For example, in the multicenter MENAC trial (which combined nutritional counseling, omega-3 fatty acid supplementation, aerobic/strength exercise, and an anti-inflammatory), patients in the intervention group maintained their weight over 6 weeks (+0.05 kg on average), while those in standard care lost nearly 1 kg (79). This combined approach effectively halted weight loss, although both groups experienced a slight decline in lean mass without significant differences (79). A 2024 review identified a greater benefit in increasing lean mass with physical exercise in cachectic versus well-nourished patients (36% vs ~12%). Studies on well-nourished patients reported an increase in lean mass with exercise in about 12% of cases, whereas this proportion rose to 36% in studies conducted on patients with weight loss (80). Regarding muscle strength, about 80% of studies report improvements (e.g., grip strength, limb strength) in exercise groups (78). Physical training, particularly resistance exercise (weights), increases the strength of trained muscle groups in cachectic patients (78). This is a significant finding, as muscle strength is correlated with functional autonomy and can contribute to improving tolerance to oncological treatments.

#### 4.1.2 Quality of life

The impact of physical exercise on the quality of life (QoL) in patients with cancer cachexia is a critical aspect, though findings in the literature are less consistent. A 2023 systematic review reported that only 38% of the QoL indicators considered showed an improvement trend with exercise (78). Many studies, due to small sample sizes, short follow-up durations, or the use of generic instruments with low sensitivity to short-term changes, did not find significant differences in QoL scores between exercise and control groups. Bozzetti (80) observed a perceptible benefit in well-being (e.g., improved appetite, energy levels, mood, and a sense of control over the disease) in 100% of malnourished/cachectic patients, compared to 47% in studies involving non-malnourished patients (80). Reinforcing this insight, other RCTs have reported significant improvements in specific QoL domains, such as physical functioning and emotional well-being.

#### 4.1.3 Systemic inflammation

Regular physical exercise, through the release of anti-inflammatory myokines (e.g., IL-10, IL-1ra), can modulate the immune response and counteract excess harmful cytokines. Although few studies have specifically measured the impact of exercise on inflammatory markers in cachectic patients, indirect evidence suggests a benefit. A meta-analysis of 83 studies (n = 3769 participants, including RCTs and controlled studies) demonstrated that regular physical activity significantly reduces circulating CRP levels, used as an indicator of systemic inflammation (81). Notably, regular aerobic exercise over several weeks led to reductions in CRP levels, independent of initial nutritional status, weight loss, or concomitant therapies (81). In patients with advanced cancer engaging in moderate exercise, a favorable trend was observed in inflammatory markers, with stabilization or slight reduction of IL-6 and CRP levels compared to sedentary controls (though results did not always reach statistical significance due to small sample sizes). In animal models of cachexia, exercise has been shown to attenuate the inflammatory cascade and mitigate weight loss induced by elevated IL-6 concentrations (82).

#### 4.1.4 Impact on survival

Counteracting cachexia through exercise could translate into improved survival, but clinical evidence is currently inconclusive. Many available studies have focused on short- to medium-term outcomes (function, weight, QoL) and were not powered to detect survival differences. To date, no RCT has definitively demonstrated a survival benefit from exercise in cachectic patients. In the study by Solheim et al. (2017) (83) (a multimodal intervention including exercise, nutrition, and anti-inflammatory treatment in patients with advanced lung or pancreatic cancer), median survival was similar between the two study arms (83). Similarly, the long-term follow-up of the MENAC trial (with full results expected in 2025) will clarify whether the combined intervention impacts mortality (79). It is important to note that existing studies may lack sufficient statistical power or duration to detect survival differences, especially given the multifactorial nature influencing outcomes in advanced cancer patients. On the other hand, robust epidemiological evidence in oncological patients (particularly with non-metastatic disease) indicates that exercising after diagnosis is associated with longer survival, as seen in breast, colon, and prostate cancers (84). Therefore, maintaining an active lifestyle in cancer patients may enhance resilience and treatment tolerance, with potential benefits for life expectancy. Current guidelines encourage physical activity in all cancer survivors and patients, although they state that further research is needed to confirm a causal effect on overall survival in advanced settings (85).

### 4.2 Exercise protocols and differences by tumor type or disease stage

Clinical studies on cachectic patients have utilized multimodal exercise programs divided into 2–3 sessions per week, adapted to the patient’s clinical status. Sessions could include:

- Moderate-intensity aerobic exercises, such as brisk walking, cycling on a stationary bike, or treadmill exercise, aimed at improving cardiorespiratory capacity and fatigue resistance, lasting approximately 20–30 minutes per session.
- Strength or resistance exercises, often using free weights, machines, elastic bands, or bodyweight (e.g., functional exercises like standing from a chair, bodyweight squats, wall push-ups), targeting major muscle groups twice a week.
- Combined programs, integrating both aerobic and strength training in the same week (e.g., two aerobic sessions + one strength session, or mixed circuits in a single session).

Some trials involved supervised sessions in hospitals or gyms under the guidance of physiotherapists or exercise physiologists, ensuring appropriate intensity and patient safety. Others favored home-based programs, providing minimal equipment (e.g., pedal exercisers, resistance bands) and instructions, with periodic monitoring. Data suggest that adherence and effectiveness are higher in structured, supervised programs (80). For instance, training with a coach allows reaching higher intensities (as in high-intensity interval training, HIIT) that could maximize anti-cachectic adaptations (80). Guidelines also recommend individualizing programs according to the patient’s functional and clinical status (considering factors such as fatigue, cardiopulmonary comorbidities, pain, anemia, etc.), gradually increasing exercise volume if tolerated.

Clinical exercise studies in cachectic patients have predominantly involved lung, pancreatic, gastrointestinal (e.g., colon, stomach), and head and neck cancers. Data comparisons reveal no marked differences in exercise efficacy based on tumor type. Overall, benefits (improvements in strength, functionality, etc.) have been observed across various advanced oncological diseases, suggesting that exercise acts on common cachexia mechanisms (muscle catabolism, physical deconditioning) rather than tumor-specific factors. Regarding cachexia severity, studies suggest that even moderately malnourished patients can effectively adhere to and benefit from exercise programs. Bozzetti (80) report no substantial differences between well-nourished and malnourished patients in adherence (~70% compliance in both groups) or in outcomes achieved with exercise (80).

## 5 Supportive medical therapies and artificial nutrition

### 5.1 Pharmacological supportive therapies

#### 5.1.1 Progestins (megestrol acetate)

Numerous studies demonstrate that megestrol acetate, at doses above 160 mg/day, can promote weight gain in cancer patients. In a randomized study, treated patients showed a significant weight increase compared to the placebo group (86–88). However, a meta-analysis indicated that although there is a trend towards weight gain, the effect is modest and not always statistically significant (89, 90). Megestrol acetate has proven effective in improving appetite even at a dose of 160 mg/day. Treated patients reported increased appetite and higher food intake compared to the placebo group (87, 88, 91). However, appetite improvement does not always translate into significant weight gain (90, 92). High doses of the drug have been associated with increases in both fat and lean mass, while lower doses have not shown relevant changes (86, 89). Some studies also report improvements in quality of life, although evidence is conflicting due to methodological limitations and small sample sizes (88, 90, 93). The drug is generally well tolerated, but high doses can lead to adverse effects such as thromboembolic events, fluid retention, adrenal insufficiency, and hypogonadism (89, 94).

#### 5.1.2 Corticosteroids

Corticosteroids are used to alleviate symptoms of cancer cachexia, particularly by stimulating appetite. However, their effects are generally short-lived and may cause side effects. They are commonly used to improve appetite and well-being in advanced-stage patients, with short-term benefits in reducing anorexia and cachexia symptoms (91, 95, 96). Weight gain usually involves both lean and fat mass (91, 95), but no significant long-term improvements in caloric intake or nutritional status have been observed (96). Therefore, corticosteroids are recommended for limited periods due to potential adverse effects and the lack of sustained benefits (13). Current guidelines recommend their use for short-term management of cancer cachexia symptoms, especially when rapid appetite stimulation is needed (5, 6).

#### 5.1.3 IL-6 (tocilizumab) and TNF-α inhibitors

Some studies have found a correlation between elevated IL-6 levels and the severity of cachexia and poor prognosis in patients with lung cancer (97, 98). Tocilizumab, an anti-IL-6 receptor antibody, has demonstrated symptom improvement in cachectic lung cancer patients overexpressing IL-6. Studies in murine models showed reduced weight loss, increased food and water intake, and prolonged survival (97). In patients with non-small cell lung cancer (NSCLC) and inflammatory cachexia, tocilizumab treatment resulted in weight gain, higher albumin levels, and reduced inflammatory markers such as C-reactive protein (98). Patients treated with tocilizumab exhibited longer median survival compared to those receiving only antitumor therapy, as well as improved appetite and reduced fatigue, leading to enhanced quality of life (98). Although treatment was generally well tolerated, adverse events such as neutropenia and skin infections were reported, although their incidence was lower than in the control group (98).

Regarding TNF-α, its role in cachexia pathogenesis has prompted research into the potential benefits of its inhibition. Thalidomide has shown promising results in treating cachexia, particularly in patients with HIV, tuberculosis, and advanced pancreatic cancer, promoting weight gain and muscle mass increase (99, 100). Sophocarpine and Matrine, which inhibit TNF-α and IL-6 production, reduced cachexia symptoms in animal models (101). Additionally, preventive administration of Adalimumab in a tumor cachexia model significantly mitigated symptoms, preserving muscle mass and reducing inflammation (102). However, the overall effectiveness of TNF-α inhibition is limited due to the complex interaction of various cytokines involved in cachexia. Further research is needed to explore combined therapies and other involved pathways.

#### 5.1.4 Cannabinoids

Some studies suggest that cannabinoids may stimulate appetite, but available evidence is conflicting and of limited quality. Meta-analyses have not found significant benefits over placebo in improving appetite or promoting weight gain in cachexia patients (103, 104). In cancer patients with cachexia, cannabinoids have not shown superior effects compared to placebo or other treatments in improving appetite or quality of life (105, 106). Some studies have even reported a deterioration in quality of life, likely due to side effects (103, 104). Although cannabinoids have potential due to their appetite-stimulating properties, current evidence does not support their widespread use in cachexia treatment.

#### 5.1.5 Androgens

Androgens, including testosterone and selective androgen receptor modulators (SARMs), show potential in treating cancer cachexia by helping to maintain or increase lean mass, particularly in hormone-insensitive tumors (107). These compounds may help alleviate symptoms by preserving muscle mass and stimulating appetite. Androgen treatment has been shown to significantly improve appetite and weight in terminal cachexia patients, with fewer side effects than megestrol acetate (108). However, another study demonstrated lower efficacy in improving appetite compared to megestrol acetate (91). SARMs are being investigated for their anabolic effects with minimal side effects, showing promising results in early clinical trials (108, 109). However, androgen therapy is contraindicated in hormone-sensitive cancers such as prostate, breast, and certain gynecological cancers due to the risk of disease progression (107).

#### 5.1.6 Antipsychotics

Recent studies indicate that olanzapine may improve appetite and promote weight gain in chemotherapy patients. In a randomized controlled trial, patients treated with olanzapine showed over 5% weight gain and improved appetite compared to the placebo group (110–112). Patients also reported improved quality of life and nutritional status, with minimal side effects, making olanzapine a well-tolerated option for managing cancer cachexia (111). Side effects observed were generally mild, such as drowsiness (113). Based on this evidence, international guidelines (5, 6) recommend its use in treating cancer cachexia.

#### 5.1.7 Formoterol

Formoterol, a long-acting β2 agonist, may play an important role in preserving muscle mass in cachectic patients. By activating β2-adrenergic receptors on skeletal muscles, it promotes protein synthesis and reduces protein degradation. Formoterol has demonstrated significant anticachectic effects by reducing muscle proteolysis and apoptosis in tumor-bearing animals, mainly by reducing ubiquitin-proteasome pathway activity (114). In cancer cachectic rats, formoterol reduced oxidative stress and myosin protein loss in respiratory and limb muscles, suggesting its role in decreasing protein oxidation and inflammation (115). A Phase I/II trial exploring the combination of formoterol with megestrol acetate in cachectic patients with advanced malignancy found this combination safe and well-tolerated, with significant improvements in muscle mass and function (116). Furthermore, combining formoterol with a soluble myostatin receptor (sActRIIB) in mice led to complete recovery of muscle weights and improved grip strength, without affecting tumor growth (117). However, while promising, clinical application requires further research to confirm safety and efficacy.

#### 5.1.8 Myostatin inhibitors

Myostatin, a member of the TGF-β family, is a negative regulator of muscle growth, making it a promising therapeutic target for cancer cachexia (118, 119). In murine models, combining the myostatin inhibitor peptide MID-035 with anamorelin increased muscle mass and strength, improving survival (118). Studies using myostatin-null mice and pharmacological agents like ACVR2B-Fc have shown myostatin inhibition preserves muscle mass in cancer cachexia. ACVR2B-Fc effectively prevented muscle atrophy without affecting tumor growth (120). Specific antibodies like PF-354 also countered muscle atrophy and improved muscle function in tumor-bearing mice (121). Soluble receptor antagonists like sActRIIB improved muscle mass and physical performance (122). However, some studies suggest myostatin inhibition alone may be insufficient, indicating the need for combined therapies targeting multiple biological pathways for optimal outcomes (123).

### 5.2 Emerging pharmacological therapies

#### 5.2.1 Anamorelin

Anamorelin, a ghrelin receptor agonist, has demonstrated the ability to improve muscle mass and stimulate appetite in cancer cachexia patients (124). Clinical studies report an increase in lean body mass ranging from 1.15 kg to 1.89 kg after 12 weeks of treatment, while placebo groups often experienced lean mass loss (125–127). Patients treated with anamorelin reported improved quality of life, general condition, and physical performance (125, 127, 128). Anamorelin demonstrated a safety profile comparable to placebo, with common side effects including fatigue and asthenia, and no significant increase in severe adverse events (126, 127). However, in patients with advanced pancreatic cancer and severe weight loss, weight gain was not significant, and rare cardiac adverse events, including fatal arrhythmias, were reported (125, 129).

#### 5.2.2 Espindolol

Espindolol, a non-selective beta-blocker, may counteract muscle depletion through its action on central β and 5-HT1α receptors, offering pro-anabolic, anti-catabolic, and appetite-stimulating effects (130). The ACT-ONE trial assessed its effectiveness in preventing weight loss and improving muscle strength in patients with NSCLC or colorectal cancer cachexia (130, 131). Although survival and weight outcomes were promising, differences were not statistically significant (130). A network meta-analysis indicated that espindolol contributed to significant weight gain, although less than olanzapine (112). Further research is needed to confirm long-term safety and efficacy (112).

#### 5.2.3 Monoclonal antibodies against GDF-15

Growth Differentiation Factor 15 (GDF-15) is involved in anorexia and weight loss common in cancer cachexia. Blocking GDF-15 is a promising strategy (132–134). Ponsegromab, a monoclonal antibody targeting GDF-15, showed promising results in early trials, improving body weight and activity levels in cancer patients (135). A Phase 2 trial showed significant weight gain and appetite improvements with higher dosages. Future Phase 3 trials will aim to confirm its efficacy and safety (11).

The main trials analyzing pharmacological approaches to cancer cachexia are summarized in **Table 1**.

**Table 1:** Pharmacological Treatment of Cancer Cachexia.

Clinical Trial	Drug/Treatment	Mechanism	Study Type	Patients (n)	Underlying Disease	Stage	Intervention Duration	Endpoints	Results
PROSPERO CRD42022358849 (90)	Megestrol acetate 320 mg/day	Hormone - stimulates appetite through direct and indirect pathways, modulates glucocorticoid activity, reduces proinflammatory cytokines, and increases food intake via neuropeptide Y release	Meta-analysis	NA	Cancer patients with anorexia–cachexia syndrome	Any	<8 weeks	Weight gain, appetite, fatigue, QoL	MA supplementation might improve QoL. No significant effect on weight, appetite, or fatigue.
ACTRN12608000405314 (93)	Megestrol acetate 480 mg vs dexamethasone 4 mg vs placebo daily	Hormonal modulation	RCT	190	Lung, gastrointestinal, and prostate cancer	Palliative care	2 weeks	Weight gain, appetite, QoL	No significant difference in weight, appetite, and QoL.
Tocilizumab (98)	Tocilizumab	Blocks inflammatory pathways mediated by interleukin-6	RCT	49	NSCLC	NA	12 weeks	Overall Survival (OS)	Longer median OS (15.1 vs. 3.2 months). Improved weight, albumin, CRP, and Glasgow Prognostic Score.
ROMANA 1, 2, 3 (125; 127)	Anamorelin 100 mg/day	Ghrelin receptor agonist	Phase 3	900	NSCLC patients	NA	12–24 weeks	Median change in lean body mass and handgrip strength	Increase in body weight, lean mass, improved QoL, no significant muscle strength improvement.
ACT-ONE (130, 131)	Espindolol (high/low dose)	Beta-blocker preventing cardiac and muscle wasting	Phase 2	87	NSCLC or colorectal cancer	Stages III or IV	16 weeks	OS and handgrip strength	Improved survival and muscle strength.
Ponsegromab (11)	Ponsegromab 100/200/400 mg every 4 weeks	Growth/differentiation factor-15 blockade	Phase 2	187	NSCLC, pancreas, colon cancer	NA	12 weeks	Weight gain	Improved body weight, appetite, and cachexia symptoms.
MENAC (NCT02330926)	Exercise routine, nutritional supplementation, and anti-inflammatory	Multimodal intervention	Phase 3	NA	Lung, pancreatic cancer (stage III/IV), NSCLC	Karnofsky Performance Status >70	6 weeks	Changes in body weight	Active. Not recruiting.

### 5.3 Enteral and parenteral nutrition

Artificial nutrition, both enteral and parenteral, can contribute to achieving a positive energy balance, improving body weight, and enhancing functional status in cancer patients with cachexia (136, 137). This approach is particularly beneficial when oral intake is insufficient (136, 138). Nutritional interventions, especially when they include high-quality nutrients, can slow the progression of cachexia and reduce muscle mass loss (21, 25). The effectiveness of artificial nutrition largely depends on the stage of cachexia at the start of treatment: patients in pre-cachexia or early-stage cachexia tend to have better outcomes than those in advanced or refractory stages (137). Therefore, personalized nutritional strategies aimed at maintaining or increasing lean mass and optimizing caloric intake are essential to improve survival and quality of life (74, 139).

#### 5.3.1 Home enteral nutrition

Home enteral nutrition can extend survival and help maintain or improve functional status in a significant proportion of patients. One study reported that 73% of patients treated with HEN survived beyond six weeks from the start of therapy, with improvements in the Karnofsky Performance Status (KPS) observed in many cases (137). Additionally, enteral nutrition proved superior to parenteral nutrition in terms of survival benefits, suggesting it should be the first choice when feasible (140). Common complications include tube blockage and spontaneous removal, but overall, this mode of nutrition is considered safe and effective in countering weight loss and improving nutritional status.

#### 5.3.2 Home parenteral nutrition

Home parenteral nutrition is reserved for advanced-stage cachectic cancer patients unable to meet their nutritional needs via oral or enteral routes. Prognosis varies significantly based on several factors, with higher KPS and lower Glasgow Prognostic Score (GPS) associated with better survival (141–143). Tumor type and extent also significantly affect prognosis, with more localized disease leading to better outcomes (142, 143). Median survival for HPN patients is typically around 3–4 months, with some studies reporting a range of 1 to 14 months (141, 144, 145). Approximately 50% survive beyond 3 months, with fewer surpassing 6 months (141, 146). Quality of life tends to remain relatively stable for those surviving beyond 3 months (145). However, HPN carries a risk of complications, notably catheter-related infections (147). Thus, HPN may be beneficial for patients with a life expectancy over 3 months, contributing to maintaining nutritional status and quality of life (145, 147).

## 6 Discussion

Optimal management of cachexia in gastrointestinal cancers requires a multipronged, personalized therapeutic approach combining nutritional interventions, physical activity, and pharmacological treatments. This integration of tailored diet, targeted exercise, and specific medical treatments is crucial for effectively addressing cachexia (2, 6). Early multidisciplinary intervention, as recommended by international guidelines, can significantly enhance both quality of life and clinical outcomes (3, 13).

There is a pressing need for large-scale randomized clinical trials to validate optimal combinations of nutritional and exercise interventions (79). Given the variability in individual patient characteristics, adopting personalized approaches based on tumor type and individual response is essential (148). Therapeutic decisions should be tailored to each case, considering disease stage, functional status, and patient symptoms. This may include adjusting caloric and protein intake, selecting targeted supplements (such as amino acids or essential fatty acids), and defining an exercise program compatible with the patient’s capacities and limitations.

Recent scientific evidence confirms the beneficial role of targeted physical exercise within this integrated framework. Studies indicate that appropriately adapted physical activity is safe, feasible, and potentially beneficial for cancer patients with cachexia (78, 148). Documented benefits include maintaining body mass (stable weight and reduced muscle loss) and improving muscle strength, with positive effects on functional capacity and various aspects of quality of life (79). Structured exercise programs can slow cachexia progression, stabilize weight, and enhance overall physical condition, such as reducing fatigue and increasing exercise tolerance (81). Physical activity may also exert beneficial effects on systemic inflammation, though this requires further investigation (82).

Several multidisciplinary clinical trials have been initiated to evaluate the impact of such integrated therapies. A notable example is the MENAC multicenter trial, combining nutritional counseling, omega-3 supplementation, physical exercise (aerobic and strength training), and anti-inflammatory medication to counter cachexia in cancer patients (83). Preliminary results are encouraging: after six weeks, patients receiving the combined intervention maintained a stable weight (+0.05 kg on average), while those on standard care lost nearly 1 kg (83). However, both groups experienced slight reductions in lean mass, with no significant differences between them, suggesting that while weight loss can be stabilized, sarcopenia may require longer durations or additional measures for significant improvement.

The long-term impact of integrated interventions on survival remains a debated issue. So far, no controlled study has conclusively demonstrated a significant prolongation of life due to anti-cachexia therapies (83). Methodological limitations, such as small sample sizes and short follow-up durations, complicate survival benefit assessments (83). The long-term follow-up of the MENAC trial, expected by 2025, will be crucial to determine whether this integrated multimodal approach can positively impact survival outcomes.

Nutritional support must also be tailored to individual needs and preferences, such as adjusting caloric and protein density, or introducing specific supplements based on identified deficits. Pharmacological strategies can complement the integrated approach: orexigenic agents (like anamorelin or megestrol acetate) can help counteract anorexia and weight loss, while anti-inflammatory or metabolic-modulating drugs (like short-term corticosteroids or novel anabolic agents) may target systemic components of cachexia. Innovative therapies, such as the monoclonal antibody ponsegromab targeting GDF-15, have shown promising initial results, significantly increasing body weight in cachectic patients with a favorable safety profile (Garcia et al., 2022). Ongoing clinical trials aim to confirm the efficacy and safety of such pharmacological approaches by 2025, potentially integrating them into multimodal protocols to improve quality of life and survival (79).

Meanwhile, emphasis remains on individualizing therapeutic pathways and integrating these measures into daily clinical practice. Physical activity should be considered and personalized for each cancer patient with cachexia whenever clinically feasible (13). Oncological and palliative societies have begun including exercise recommendations in their guidelines, recognizing that appropriately monitored exercise is safe and functionally beneficial even in advanced disease (2). Training programs must be tailored to individual capabilities; even mild-to-moderate physical activity, if performed consistently, can help maintain strength, autonomy, and daily functioning (78).

In conclusion, future perspectives in managing cancer cachexia aim towards increasingly personalized and integrated care. Combining targeted nutritional interventions, adapted physical activity programs, and innovative pharmacological therapies seeks to address cachexia on all fronts, offering comprehensive support to gastrointestinal cancer patients. The ultimate goal is to enhance clinical status, functionality, and quality of life, while potentially influencing disease progression positively. The current challenge for the scientific community is to validate these multimodal strategies through robust clinical trials, ultimately translating them into standardized clinical recommendations and protocols that ensure optimal therapeutic opportunities for every cachectic patient.

## 7 Conclusions

The management of cancer cachexia in patients with gastrointestinal tumors requires a complex and personalized approach that integrates nutrition, physical activity, and pharmacological therapies. Current evidence supports the role of a high-protein, high-calorie diet, with particular attention to the integration of omega-3 fatty acids, branched-chain amino acids, and other nutraceuticals that may modulate the inflammatory response and muscle metabolism. However, the effectiveness of nutrition alone is limited in advanced stages of cachexia, emphasizing the need for combined strategies.

Physical activity emerges as a crucial element in cachexia management, with evidence demonstrating improvements in muscle mass, strength, and quality of life. Resistance and combined exercises appear to be the most effective in reducing muscle catabolism and improving physical functionality, although further research is needed to define the optimal protocol for cancer patients in advanced conditions.

Parallelly, various drugs have shown potential in managing cachexia, ranging from progestins to inflammatory cytokine inhibitors like tocilizumab, to more recent ghrelin agonists and muscle metabolism modulators. However, patient responses to these therapies vary, and there is no consensus on the most effective combination. International guidelines suggest a multidisciplinary approach that integrates nutritional interventions, physical exercise, and pharmacotherapy, with continuous monitoring of nutritional and functional status.

Despite progress, numerous clinical challenges remain. The lack of large-scale randomized studies limits the clinical applicability of many proposed strategies. Additionally, individual variability in treatment response makes personalized approaches essential, based on thorough patient assessment. Future studies should focus on identifying predictive biomarkers for multimodal intervention responses and validating integrated strategies to optimize cancer cachexia management in gastrointestinal tumors.
